# Perilipin 5 fine-tunes lipid oxidation to metabolic demand and protects against lipotoxicity in skeletal muscle

**DOI:** 10.1038/srep38310

**Published:** 2016-12-06

**Authors:** Claire Laurens, Virginie Bourlier, Aline Mairal, Katie Louche, Pierre-Marie Badin, Etienne Mouisel, Alexandra Montagner, André Marette, Angelo Tremblay, John S. Weisnagel, Hervé Guillou, Dominique Langin, Denis R. Joanisse, Cedric Moro

**Affiliations:** 1 INSERM, UMR1048, Institute of Metabolic and Cardiovascular Diseases, Toulouse, France; 2 University of Toulouse, Paul Sabatier University, France; 3 INRA, UMR 1331, TOXALIM, Toulouse, France; 4 Department of Medicine, Laval University, Quebec City, Canada; 5 Centre de Recherche de l’Institut Universitaire de Cardiologie et de Pneumologie de Québec, Laval University, Quebec City, Canada; 6 Department of Kinesiology, Laval University, Quebec City, Canada; 7 CHU-CHUQ, Laval University, Quebec City, Canada; 8 Toulouse University Hospitals, Department of Clinical Biochemistry, Toulouse, France

## Abstract

Lipid droplets (LD) play a central role in lipid homeostasis by controlling transient fatty acid (FA) storage and release from triacylglycerols stores, while preventing high levels of cellular toxic lipids. This crucial function in oxidative tissues is altered in obesity and type 2 diabetes. Perilipin 5 (PLIN5) is a LD protein whose mechanistic and causal link with lipotoxicity and insulin resistance has raised controversies. We investigated here the physiological role of PLIN5 in skeletal muscle upon various metabolic challenges. We show that PLIN5 protein is elevated in endurance-trained (ET) subjects and correlates with muscle oxidative capacity and whole-body insulin sensitivity. When overexpressed in human skeletal muscle cells to recapitulate the ET phenotype, PLIN5 diminishes lipolysis and FA oxidation under basal condition, but paradoxically enhances FA oxidation during forskolin- and contraction- mediated lipolysis. Moreover, PLIN5 partly protects muscle cells against lipid-induced lipotoxicity. In addition, we demonstrate that down-regulation of PLIN5 in skeletal muscle inhibits insulin-mediated glucose uptake under normal chow feeding condition, while paradoxically improving insulin sensitivity upon high-fat feeding. These data highlight a key role of PLIN5 in LD function, first by finely adjusting LD FA supply to mitochondrial oxidation, and second acting as a protective factor against lipotoxicity in skeletal muscle.

Cytosolic lipid droplets (LD) are important energy-storage organelles in most tissues^1^. LD are composed of a lipid core, mainly made of triacylglycerols (TAG), surrounded by a phospholipid monolayer in which are embedded proteins^2^,^3^. LD are dynamic organelles playing a central role in fatty acid (FA) trafficking^4^. Importantly, it has been suggested that altered LD dynamics could contribute to the development of muscle insulin resistance, by facilitating the emergence of cellular toxic lipids such as diacylglycerols (DAG) and ceramides (CER) known to impair insulin action^5^,^6^. LD therefore buffers intracellular FA flux, a function particularly critical in oxidative tissues such as skeletal muscle with a high lipid turnover and metabolic demand^7^. Skeletal muscle is also a main site for postprandial glucose disposal, and muscle insulin resistance is a major risk factor of type 2 diabetes^8^.

The LD surface is coated by perilipins and other structural proteins^1^. Enzymes involved in lipid metabolism such as lipases and lipogenic enzymes interact with LD. Perilipin 5 (PLIN5) belongs to the family of perilipins, and is highly expressed in oxidative tissues such as liver, heart, brown adipose tissue and skeletal muscle^9^,^10^. A recent study from Bosma and colleagues has described that overexpressing PLIN5 in mouse skeletal muscle increases intramyocellular TAG (IMTG) content^11^, which is in agreement with other studies showing that PLIN5 acts as a lipolytic barrier to protect the LD against the hydrolytic activity of cellular lipases^12^,^13^. Interestingly, PLIN5 was also described to localize to mitochondria^14^, and suggested to enhance FA utilization^15^. However, a protective role of PLIN5 against lipid-induced insulin resistance could not be confirmed after gene electroporation of PLIN5 in rat *tibialis anterior* muscle^11^ and muscle-specific PLIN5 overexpression in mice^16^. In addition, a direct role of PLIN5 in facilitating FA oxidation upon increased metabolic demand has never been demonstrated in skeletal muscle.

To reconcile data from the literature, a hypothetical model would be that PLIN5 exhibits a dual role, buffering intracellular FA fluxes to prevent lipotoxicity in the resting state on one hand, and facilitating FA oxidation upon increased metabolic demand in the contracting state on the other hand. The aim of the current work was therefore to investigate the putative dual role of PLIN5 in the regulation of FA metabolism in skeletal muscle. The functional role of PLIN5 was studied *in vitro* in human primary muscle cells and *in vivo* in mouse skeletal muscle. Our data here reveal a key role of PLIN5 to adjust LD FA supply to metabolic demand, and also demonstrate that changes in PLIN5 expression influences lipotoxicity and insulin sensitivity in skeletal muscle.

## Results

### PLInN5 relates to oxidative capacity in mouse and human skeletal muscle

**Figure 1:**
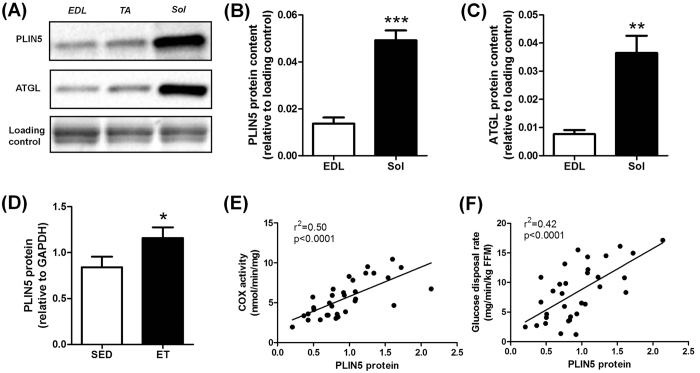
PLIN5 relates to oxidative capacity in mouse and human skeletal muscle. Representative blots (**A**) and quantification of PLIN5 (**B**) and ATGL (**C**) protein content in different mouse skeletal muscles (n = 5) (EDL: *extensor digitorum longus*, TA: *tibialis anterior*, Sol: *soleus*). **p < 0.01, ***p < 0.001 versus EDL. (**D**) Quantification of PLIN5 protein content in *vastus lateralis* muscle of healthy lean and endurance-trained volunteers (n = 11 per group). Correlations between muscle PLIN5 protein and (**E**) cytochrome oxidase activity, and (**F**) glucose disposal rate (n = 33). *p < 0.05 versus lean.

Muscle PLIN5 content was measured in various types of skeletal muscles in the mouse (Fig. 1A). We observed that PLIN5 was highly expressed in oxidative *soleus* muscle compared to mixed *tibialis anterior* or to the more glycolytic *extensor digitorum longus* muscle (3.6 fold, p < 0.001) (Fig. 1B). A similar expression pattern was observed for ATGL protein (4.7 fold, p = 0.0019) (Fig. 1C). In human *vastus lateralis* muscle, we observed a higher PLIN5 protein content in lean endurance-trained compared to lean sedentary individuals (+38%, p = 0.033) (Fig. 1D). A robust relationship between muscle PLIN5 and cytochrome oxidase activity, a marker of muscle oxidative capacity, was observed (r^2^ = 0.50, p < 0.0001) (Fig. 1E). Significant positive correlations were also noted with citrate synthase activity (r^2^ = 0.42, p < 0.0001) and β-hydroxy-acyl-CoA-dehydrogenase (r^2^ = 0.23, p = 0.0053). Importantly, muscle PLIN5 protein show a strong positive association with glucose disposal rate measured during euglycemic hyperinsulinemic clamp in subjects with varying degrees of BMI and fitness (r^2^ = 0.42, p < 0.0001) (Fig. 1F). Collectively, these data show that PLIN5 relates to muscle oxidative capacity and insulin sensitivity in mouse and human skeletal muscle.

### PLIN5 overexpression reduces lipolysis and FA oxidation under basal connditions in human primary myotubes

**Figure 2:**
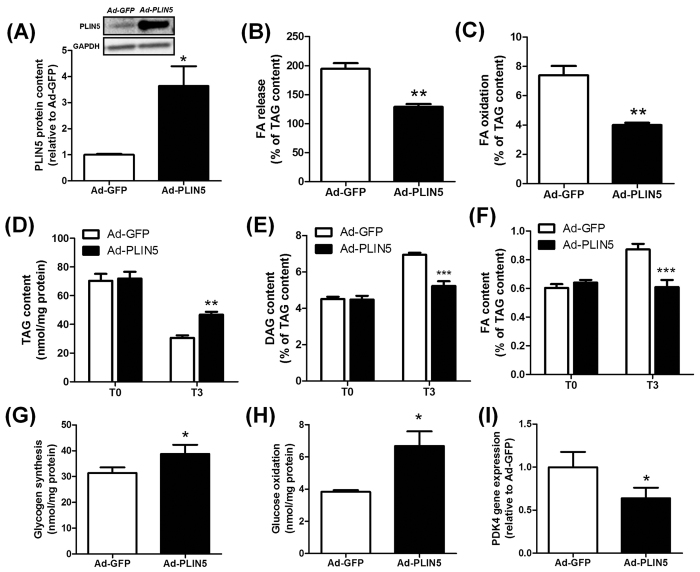
PLIN5 overexpression reduces lipolysis and FA oxidation under basal conditions in human primary myotubes. (**A**) Representative blot and quantification of PLIN5 protein content in control (Ad-GFP) and PLIN5-overexpressing myotubes (Ad-PLIN5) (n = 3). Pulse-Chase studies using [1-^14^C] oleate were performed to determine (**B**) FA release into the culture medium (Ad-GFP = 59 ± 1.8 nmol/3 h/mg protein), (**C**) FA oxidation (Ad-GFP = 2.22 ± 0.13 nmol/3 h/mg protein), and the rate of incorporation of radiolabeled oleate into (**D**) TAG, (**E**) DAG (T0 Ad-GFP = 3.14 ± 0.14 nmol/3 h/mg protein) and (**F**) intracellular FA content (T0 Ad-GFP = 0.42 ± 0.03 nmol/3 h/mg protein) in control (Ad-GFP) and PLIN5-overexpressing myotubes (Ad-PLIN5). (**G**) Glycogen synthesis and (**H**) glucose oxidation were measured in control myotubes (Ad-GFP) and myotubes overexpressing PLIN5 (Ad-PLIN5) using [U-^14^C] glucose. (**I**) PDK4 gene expression was measured in control (Ad-GFP) and PLIN5-overexpressing myotubes (Ad-PLIN5). (n = 6) *p < 0.05, **p < 0.01 ***p < 0.001 versus Ad-GFP.

Human skeletal muscle cells differentiated into myotubes are suited to perform mechanistic and metabolic studies^11^. However, PLIN5 mRNA expression is nearly undetectable in human primary myotubes compared to human muscle tissue (11 Ct difference, 2^11^ = 2054 fold lower expression) (Supplemental Fig. S1). To recapitulate the ET phenotype *in vitro* in skeletal muscle cells, we overexpressed PLIN5 to gain further insight into its functional and metabolic role. Adenovirus-mediated PLIN5 overexpression led to a significant increase of PLIN5 protein content (3.6-fold, p = 0.013) (Fig. 2A). We first examined the effect of PLIN5 overexpression on lipolysis and FA metabolism under basal condition, using a Pulse-Chase design. Endogenous TAG pool was pre-labeled (i.e. pulsed) overnight using [1-^14^C] oleate. At the end of the pulse phase (i.e. T0), cells were chased for 3 h in a medium containing a low glucose concentration to promote lipolysis (i.e. T3). We found that PLIN5 overexpression decreased FA release into the culture medium (−34%, p = 0.044) (Fig. 2B), which was accompanied by a sharp reduction of FA oxidation compared to control cells (−46%, p = 0.0025) (Fig. 2C). We observed a 56% TAG depletion at T3 (i.e. after 3 hours of chase in a low-glucose medium) in control cells. PLIN5 overexpressing cells exhibited a lower TAG depletion rate compared to control cells (−38%, p = 0.0022) (Fig. 2D). Since the size of the TAG pool is a major determinant of TAG breakdown rate^17^, lipid trafficking rates (FA and DAG) were normalized to TAG content. Consistently, intracellular DAG and FA accumulation during the chase period was totally abrogated by PLIN5 overexpression (Fig. 2E,F).

FA and glucose are the main nutrients competing for fuel oxidation in skeletal muscle^18^. By slowing down lipid utilization, PLIN5 overexpression enhanced basal glycogen synthesis (+24%, p = 0.045) (Fig. 2G) and glucose oxidation (+74%, p = 0.010) (Fig. 2H). As previously observed in this cell model system^19^, this metabolic switch was paralleled by a significant down-regulation of *pyruvate dehydrogenase kinase 4* (PDK4) (−36%, p = 0.024) (Fig. 2I). Taken together, these results clearly show that PLIN5 overexpression slows down lipolysis and FA oxidation and favors a switch towards glucose metabolism in human muscle cells.

### PLIN5 overexpression facilitates lipid oxidation upon increased metabolic demand

**Figure 3:**
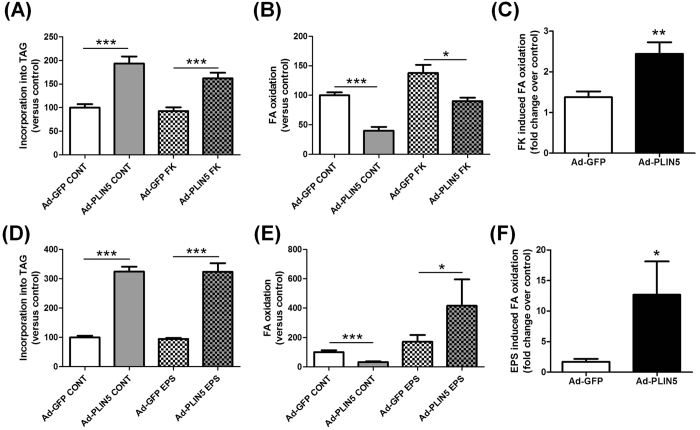
PLIN5 overexpression facilitates lipid oxidation upon increased metabolic demand. Pulse-Chase studies using [1-^14^C] oleate were performed to determine the rate of (**A,D**) incorporation of radiolabeled oleate into TAG and (**B,E**) oleate oxidation in control myotubes (Ad-GFP) and myotubes overexpressing PLIN5 (Ad-PLIN5) either during (**A–C**) forskolin (FK) (Ad-GFP CONT = 1.66 ± 0.25 nmol/3 h/mg protein) or (**D–E**) electrical pulse (EPS) stimulation (Ad-GFP CONT = 6.33 ± 2.05 nmol/24 h/mg protein) (n = 6). Values are expressed in % of Ad-GFP Control (**A,B,D,E**) and in fold change over control in (**C** and **F**). *p < 0.05, **p < 0.01 ***p < 0.001 versus Ad-GFP.

Considering that PLIN5 is elevated in skeletal muscle of athletes with a high lipid turnover, we investigated its role under stimulation of lipolysis, increased TAG turnover and metabolic demand in human primary myotubes. Thus PLIN5-mediated TAG accumulation (+93%, p = 0.0002) was reduced in the presence of forskolin (a potent lipolysis activator) (+75%, p = 0.0006) (Fig. 3A). In line with this, FA oxidation was reduced by 60% in PLIN5 overexpressing myotubes under basal conditions (p < 0.0001), while this decrease was of only 35% upon forskolin stimulation (p = 0.01) (Fig. 3B). Overall, PLIN5 overexpression greatly potentiated forskolin-induced FA oxidation when compared to control cells (+77%, p = 0.007) (Fig. 3C).

Because muscle contraction represents a more physiological stimulation of FA metabolism and increased metabolic demand, we used a model of electrical pulse stimulation (EPS) to recapitulate contraction-mediated lipolysis *in vitro*. As a model validation, we observed no significant change in total glycogen content and a sharp increase of FA oxidation, which represent classical skeletal muscle physiological adaptations to endurance training (REF). These effects were accompanied by a robust induction of interleukin-6 gene expression, a well-known exercise-induced myokine (Supplemental Fig. S2). Interestingly, despite no major change in TAG pools under basal or stimulated conditions (Fig. 3D), we observed a very sharp increase of EPS-mediated FA oxidation in PLIN5 overexpressing cells (2.4 fold, p = 0.014) (Fig. 3E). Importantly, we observed that EPS increased FA oxidation by 1.7 fold in control myotubes, while this effect was robustly enhanced up to 12.7 fold in PLIN5 overexpressing cells (Fig. 3F). Together, this suggests for the very first time that PLIN5 is necessary to boost TAG lipolysis and FA oxidation upon increased metabolic demand in skeletal muscle.

### PLIN5 exert a protective role against palmitate-induced lipotoxicity

**Figure 4:**
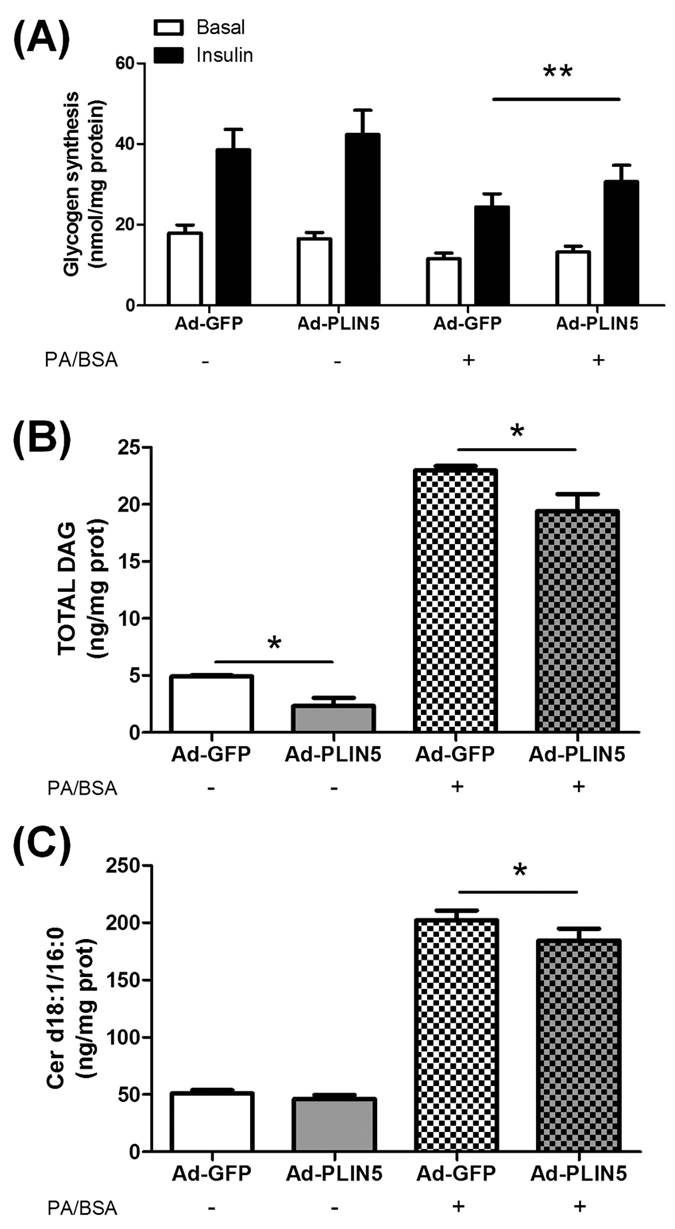
PLIN5 exerts a protective role against palmitate-induced lipotoxicity. (**A**) Glycogen synthesis was measured in control myotubes (Ad-GFP) and myotubes overexpressing PLIN5 (Ad-PLIN5) using [U-^14^C] glucose in absence or presence of 100 nM insulin, in control cells and in cells treated with 300 μM of palmitic acid for 24 h (n = 9). (**B**) Total diacylglycerols (DAG) and (**C**) Ceramide (CER) d18:1/16:0 content were measured in control myotubes (Ad-GFP) and myotubes overexpressing PLIN5 (Ad-PLIN5) (n = 4). *p < 0.05, **p < 0.01 versus Ad-GFP.

Besides a key role in controlling LD lipolysis, PLIN5 may sequester toxic lipids into LD and reduce intracellular lipotoxic insults^20^. To test this hypothesis, we challenged myotubes with palmitate at a concentration known to induce lipotoxicity and insulin resistance^21^. As a model validation, we first observed that palmitate treatment strongly elevated total diacylglycerols (+4.7 fold, p < 0.0001) and ceramides (+4 fold, p < 0.0001) levels while inhibiting insulin-mediated glycogen synthesis (−37%, p = 0.016) (Fig. 4). Of note, PLIN5 overexpressing myotubes were partly protected from palmitate-mediated insulin resistance and lipotoxicity. Insulin-stimulated glycogen synthesis was higher in PLIN5 overexpressing myotubes challenged with palmitate compared to control myotubes (+26%, p = 0.0025) (Fig. 4A). Similarly, palmitate-mediated DAG accumulation was slightly reduced in PLIN5 overexpressing myotubes (−16%, p = 0.039) (Fig. 4B). Finally, PLIN5 overexpressing myotubes displayed reduced concentrations of all ceramides species measured in response to palmitate treatment (Two-way ANOVA p = 0.04), particularly due to reduced ceramide d18:1/16:0 content (Fig. 4C), the most abundant ceramide species in our cell model. Collectively, these results highlight a slight protective role of PLIN5 against lipotoxicity and palmitate-induced insulin resistance in muscle cells.

### PLIN5 knockdown in mouse skeletal muscle increases lipid oxidation and reduces insulin-stimulated glucose uptake under normal chow diet

**Figure 5:**
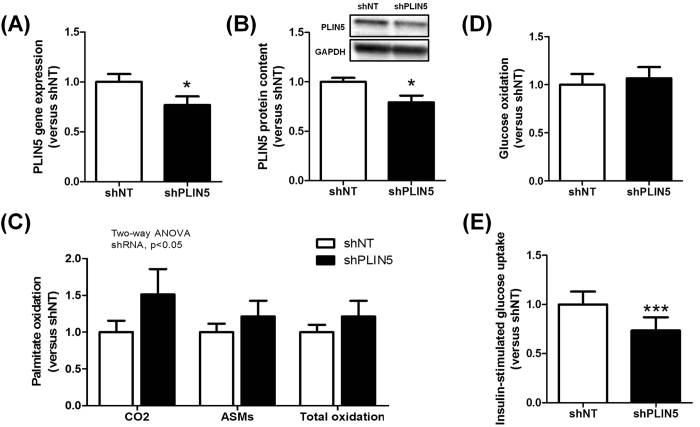
PLIN5 knockdown in mouse skeletal muscle increases lipid oxidation and reduces insulin-stimulated glucose uptake under normal chow diet. PLIN5 (**A**) gene expression and (**B**) protein content measured in control (shNT) and PLIN5 silenced (shPLIN5) mouse *tibialis anterior* muscle (n = 6). Palmitate (**C**) and glucose (**D**) oxidation rate were measured using respectively [U-^14^C] glucose or [1-^14^C] palmitate in control (shNT) and PLIN5 silenced (shPLIN5) muscle homogenates. Palmitate oxidation (i.e. CO2), acid soluble metabolites accumulation (i.e. ASMs) and total oxidation (i.e. the sum of CO2 release and ASMs accumulation) were measured (n = 6). (**E**) Insulin-stimulated glucose uptake was determined in control (shNT) and PLIN5 knockdown (shPLIN5) muscles. (n = 7). *p < 0.05, ***p < 0.001 versus shNT.

Considering that PLIN5 is strongly expressed in skeletal muscle and that previous gain-of-function studies in muscle failed to substantiate the causal and mechanistic link between PLIN5 and insulin sensitivity, we assessed the physiological role of PLIN5 *in vivo* by inducing a muscle-restricted loss-of-function. We knocked down its expression by injecting an AAV1 containing a shRNA directed against PLIN5 in *tibialis anterior* muscle of 10-week old C57BL/6 J mice. Intramuscular AAV1-shRNA-PLIN5 injection significantly reduced PLIN5 mRNA expression (−23%, p = 0.014) (Fig. 5A) and protein content (−21%, p = 0.024) (Fig. 5B) compared to the contralateral leg injected with an AAV1 containing a non-targeted shRNA. Of note, no functional compensation by other PLIN isoforms was observed in PLIN5 knocked down muscles (Supplemental Fig. S3). In agreement with *in vitro* data in the basal state, knockdown of PLIN5 increased the rate of FA oxidation to CO_2_ (+51%) and ASM (i.e. acid soluble metabolites) (+21%) (p < 0.05) (Fig. 5C). No change in glucose oxidation was observed (Fig. 5D). Since PLIN5 null mice exhibit signs of insulin resistance in skeletal muscle^22^, we next measured insulin-stimulated glucose uptake. Interestingly, PLIN5 knockdown decreased insulin-stimulated muscle glucose uptake (−27%, p = 0.0003) (Fig. 5E). However, muscle insulin resistance appeared independent of significant change in total (shNT 0.11 ± 0.01 *vs.* shPLIN5 0.12 ± 0.02 nmol/mg, NS) and various ceramide species. Taken together, our data argue for a physiological role of PLIN5 in the regulation of FA oxidation and insulin sensitivity in skeletal muscle *in vivo*.

### PLIN5 knockdown in mouse skeletal muscle ameliorates insulin action under high-fat feeding

**Figure 6:**
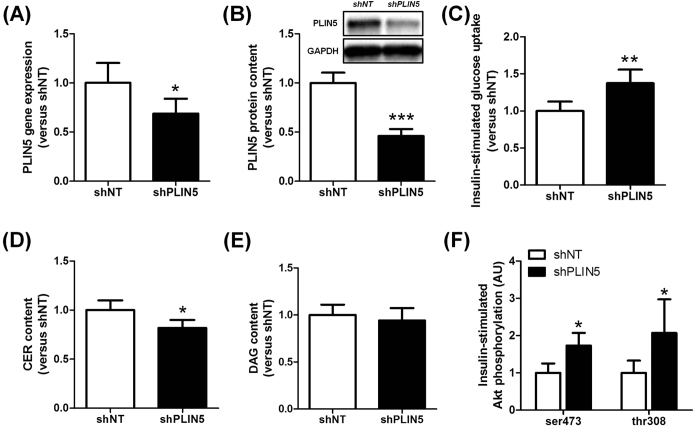
PLIN5 knockdown in mouse skeletal muscle ameliorates insulin action under high-fat feeding. PLIN5 (**A**) gene expression and (**B**) protein content measured in control (shNT) and PLIN5 silenced (shPLIN5) mouse *tibialis anterior* muscle (n = 6). (**C**) Insulin-stimulated glucose uptake, (**D**) total ceramide (CER) and (**E**) total diacylglycerols (DAG) content were determined in control (shNT) and PLIN5 knockdown (shPLIN5) muscles. (n = 7). (**F**) Insulin-stimulated Akt phosphorylation on Ser473 and Thr308 residues was measured in control (shNT) and PLIN5 silenced (shPLIN5) muscle (n = 4). *p < 0.05, **p < 0.01, ***p < 0.001 versus shNT.

We next investigated the impact of PLIN5 knockdown in *tibialis anterior* muscle under high fat diet feeding for 12 weeks. Intramuscular AAV1-shRNA-PLIN5 injection in HFD-fed mice reduced PLIN5 mRNA level by 31% (p = 0.015) (Fig. 6A) and protein content by 54% (p = 0.0003) (Fig. 6B), without any compensatory changes in the expression level of PLIN2, PLIN3 and PLIN4 (Supplemental Fig. S3). In contrast with normal chow diet-fed mice, insulin-stimulated muscle glucose uptake was improved in PLIN5 knocked down legs of HFD-fed mice (+37%, p = 0.0031) (Fig. 6C). This was accompanied by a decrease in total ceramide content (−18%, p = 0.028) (Fig. 6D), while DAG content remained unchanged (Fig. 6E). In agreement, we also observed a significant increase of insulin-stimulated Akt phosphorylation on serine 473 and threonine 308 in PLIN5 knockdown muscle compared to the contralateral leg (1.7 fold and 2.1 fold, respectively, p = 0.048) (Fig. 6F). Collectively, while PLIN5 knockdown promotes insulin resistance in skeletal muscle of chow-fed mice, it paradoxically partly protects skeletal muscle against HFD-induced insulin resistance.

### High-fat feeding up-regulates PLIN5 in skeletal muscle independently of PPARβ

PLIN5 has been described as a *Peroxisome Proliferator-Activated Receptors* (PPAR)-target gene in a mouse muscle cell line model^23^. Since we noted a striking up-regulation of PLIN5 with high-fat feeding at both mRNA and protein levels (Supplemental Fig. S4), we examined PLIN5 regulation by PPAR *in vitro* and *in vivo*. We confirmed previous findings^23^ showing that PLIN5 is a PPARβ-responsive gene in human primary myotubes (Supplemental Fig. S4). Interestingly, PLIN5 was specifically induced by a PPARβ agonist (GW0742) in this cell model system (5.3 fold, p < 0.001). We next investigated whether HFD-mediated up-regulation of PLIN5 was mediated by activation of PPARβ in skeletal muscle *in vivo*. Of interest, muscle PLIN5 protein content was similar in PPARβ knockout mice, while HFD-mediated up-regulation of PLIN5 was unaffected in PPARβ knockout mice (Supplemental Fig. S4). Thus, HFD-mediated up-regulation of PLIN5 could be seen as an adaptive response to facilitate fat storage into LD of excess incoming FA and minimize lipotoxicity. Although PLIN5 is a PPARβ-responsive gene in skeletal muscle, HFD-mediated up-regulation of PLIN5 appears independent of PPARβ.

## Discussion

**Figure 7:**
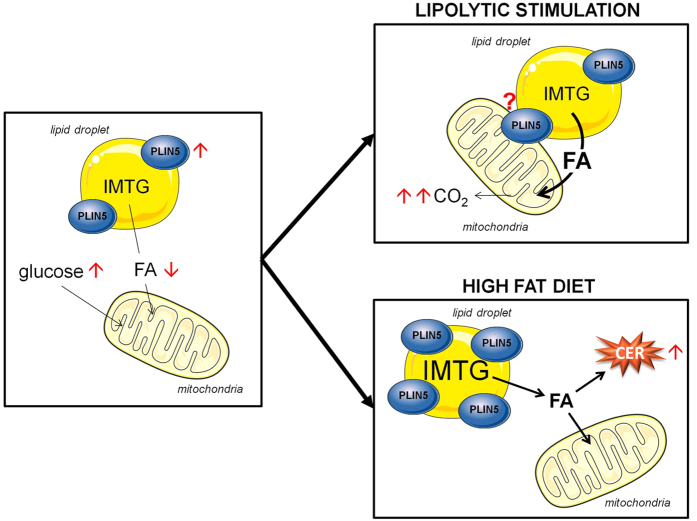
Proposed mechanistic model of PLIN5 in skeletal muscle upon various metabolic states. In the resting state, PLIN5 protects LD from lipolytic attack by lipases. An increase in PLIN5 content (red arrows) slows down lipolysis and FA oxidation, favoring a switch towards glucose utilization. During lipolytic stimulation (i.e. PKA activation or contraction), PLIN5 enhances FA oxidation, thereby increasing CO_2_ production. It has been suggested that PLIN5 could provide a physical linkage between LD and mitochondria. We can hypothesize that this relocation has metabolic consequences by facilitating FA channeling from LD to mitochondria, thus allowing a more efficient coupling between IMTG lipolysis and FA oxidation upon increased metabolic demand. Finally, the up-regulation of PLIN5 with high-fat feeding is insufficient to protect from LD-mediated CER accumulation. FA: Fatty Acids; IMTG: Intramyocellular Triacylglycerols; CER: Ceramides.

LD play a critical role in oxidative tissues to maintain appropriate fuel supply during periods of energy needs but also to buffer daily fluxes of FA to avoid cellular lipotoxicity. PLIN5 has been previously shown as a LD protein inhibiting lipolysis and correlating with insulin sensitivity^13^,^24^,^25^. The current work demonstrates for the first time that PLIN5 protects against palmitate-induced insulin resistance and facilitates FA oxidation in response to muscle contraction and increased metabolic demand *in vitro*. We further show a causal link between down-regulation of PLIN5 and insulin resistance *in vivo* in mouse skeletal muscle. We show here that the skeletal muscle enriched PLIN5 protein has a key role in controlling fat oxidation and lipotoxicity by fine tuning FA fluxes in and out of the LD from the resting to the contracting state. PLIN5 facilitates fat storage into LD and inhibits FA oxidation in the resting state while sharply boosting IMTG lipolysis and FA oxidation during muscle contraction or PKA stimulation (Fig. 7). Although the precise molecular mechanism was not investigated here, one can speculate that PLIN5 is physically relocated out of the LD to favor LD hydrolysis by adipose triglyceride lipase and FA channeling into mitochondria^15^.

We first observed that PLIN5 tightly correlates with oxidative capacity of mouse and human skeletal muscle. Muscle PLIN5 content strongly correlated as well with whole-body insulin sensitivity. In addition, we confirm that endurance-trained subjects exhibited higher levels of PLIN5 protein compared to lean sedentary subjects as previously described^24^. This is in line with various studies showing that aerobic exercise training increases PLIN5 protein, oxidative capacity and insulin sensitivity in skeletal muscle^26^,^27^,^28^. Thus endurance-trained individuals display higher lipid content, oxidative capacity and insulin sensitivity compared to matched sedentary controls^29^,^30^. We next observed that PLIN5 overexpression in human primary myotubes has a modest protective effect against saturated fat-induced lipotoxicity and insulin-resistance. Thus PLIN5 seems to preserve insulin action (glycogen synthesis) by sequestering toxic saturated lipids into LDs^31^,^32^. Our data are in agreement with a recent study showing that PLIN5 overexpression in C2C12 mouse myotubes facilitate palmitate sequestration into LD and remodels their lipid composition^20^. Finally, a recent study by Mason and colleagues reported that PLIN5 knockout mice develop insulin resistance associated with ceramide accumulation in skeletal muscle^22^.

Studies from different groups have shown that PLIN5 overexpression increases TAG storage in mouse skeletal^11^ and cardiac^12^ muscle. We observed here that PLIN5 overexpression slows down TAG-derived lipolysis and FA oxidation in basal resting conditions, and concomitantly induces a switch towards glucose utilization. Of importance, two reports described that PLIN5 not only localizes to the LD surface, but also to the mitochondria^14^,^15^. We show here for the first time that PLIN5 overexpression in human primary myotubes sharply enhanced FA oxidation upon forskolin- and contraction-induced lipolysis activation and metabolic demand. This suggests that PLIN5 might provide a physical linkage between LD and mitochondria in a context of increased energy demand. Our data are in agreement with data in ALM12 liver cells in which PLIN5 overexpression enhanced FA release when lipolysis was activated by the adenylyl cyclase activator forskolin^15^. PLIN5 appears to be phosphorylated by PKA (cAMP-dependent protein kinase)^33^, in a similar fashion as PLIN1 in adipose tissue^34^. The molecular pathways induced by contraction converging to PLIN5 where not investigated here and require a detailed examination in future studies.

We next investigated the physiological role of PLIN5 in skeletal muscle *in vivo* through loss-of-function studies using AAV gene delivery. PLIN5 was knocked down in one leg of mice fed either standard chow or high fat diets. In line with our *in vitro* data, PLIN5 knockdown induced a compensatory increase of FA oxidation rate under standard chow diet. This could be explained by a better access of lipases to LD and greater TAG turnover as shown in cardiac muscle of PLIN5-deficient mice^35^. Interestingly, it has been described that a global PLIN5 deficiency induces muscle insulin resistance^22^. However, this effect may be confounded by systemic factors. In line with the positive association between muscle PLIN5 content and insulin sensitivity observed in humans, we show that a partial down-regulation of PLIN5 in mouse skeletal muscle inhibits insulin-stimulated glucose uptake. This highlights for the first time that down-regulation of PLIN5 promotes insulin resistance in a muscle-autonomous fashion. This might be explained by a deficient coupling between FA supply and mitochondrial oxidation resulting in muscle inflammation, although further work is needed to better understand these mechanisms.

Under HFD, we first observed a striking up-regulation of PLIN5 mRNA and protein levels in skeletal muscle. We describe here PLIN5 as a PPARβ target gene in human primary skeletal muscle cells, which is in agreement with data from C2C12 mouse myotubes^23^. However, we show for the first time that baseline expression of muscle PLIN5 is not influenced by PPARβ, and that HFD-mediated up-regulation of PLIN5 is not driven by PPARβ. Although baseline expression of muscle PLIN5 is strongly reduced in PPARα knockout mice, HFD-mediated up-regulation of PLIN5 is not prevented as well in these transgenic mice^23^. It is still unclear how HFD promotes the up-regulation of muscle PLIN5 but this may be under the control of PPARγ which contribute to lipid accumulation in skeletal muscle during high fat feeding^36^. Other transcription factors related to lipid storage may be involved. Thus, HFD-mediated up-regulation of PLIN5 seems to be an adaptive mechanism to favor FA sequestering and accumulation into TAG pools. However, and contrary to our expectations, muscle PLIN5 knockdown in a context of high fat diet improved muscle insulin sensitivity. It has been previously shown that high fat feeding is accompanied by an upregulation of muscle oxidative capacity which does not appear sufficient to prevent both TAG accumulation and lipotoxicity^37^,^38^. Thus, lower levels of PLIN5 during HFD, achieved by AAV-mediated knockdown, might in this context facilitate IMTG lipolysis and FA utilization in a resting muscle with high lipid content, and therefore reduce lipotoxicity. This hypothesis is partly supported by the observation of a reduced total ceramide content and an increase of Akt Ser473 and Thr308 phosphorylation, while no change in total DAG was observed in PLIN5-knocked down muscle of HFD-fed mice. These data are consistent with at least another study showing that PLIN5 knockout mice display a markedly improved glucose tolerance under HFD with a trend toward increased peripheral glucose clearance^22^. Thus our data brings light on this previous observation showing an elevated rate of glucose uptake in PLIN5-deficient skeletal muscles. Overall, although PLIN5 exhibits a protective role against lipotoxicity in standard nutritional conditions, HFD-mediated up-regulation of PLIN5 appears deleterious for the maintenance of insulin action in skeletal muscle.

In summary, we provide mechanistic evidences that PLIN5 plays a key role in skeletal muscle. We show for the first time a dual role of PLIN5, favoring TAG accumulation and protecting from high intracellular toxic lipid levels in the resting state, while facilitating IMTG lipolysis and FA oxidation during contraction and increased metabolic demand. This work further highlights the important role of LD function and dynamics for metabolic regulation and for the maintenance of insulin sensitivity in skeletal muscle.

## Methods

### Human muscle sampling

Data and samples from men aged between 34 and 53 years with varying degree of BMI and insulin sensitivity were available from a prior study (n = 33)^39^. Of these, 11 were normal weight sedentary controls, 11 were obese sedentary and 11 were normal weight endurance-trained individuals. The overall study design and subject testing have been partly described in^39^. The study was performed according to the latest version of the Declaration of Helsinki and the Current International Conference on Harmonization (ICH) guidelines. The research protocol was approved by the Université Laval ethics committee and all subjects provided written informed consent. Samples of *vastus lateralis* (~40 mg) were obtained, blotted free of blood, cleaned to remove fat and connective tissue and snap-frozen in liquid nitrogen for Western blot analyses. All samples were stored at −80 °C under argon or nitrogen gas until use.

### Skeletal muscle primary cell culture

Satellite cells from *rectus abdominis* of healthy male subjects (age 34.3 ± 2.5 years, BMI 26.0 ± 1.4 kg/m^2^, fasting glucose 5.0 ± 0.2 mM) were kindly provided by Prof. Arild C. Rustan (Oslo University, Norway). Satellite cells were isolated by trypsin digestion, preplated on an uncoated petri dish for 1 h to remove fibroblasts, and subsequently transferred to T-25 collagen-coated flasks in Dulbecco’s Modified Eagle’s Medium (DMEM) low glucose (1 g/L) supplemented with 10% FBS and various factors (human epidermal growth factor, BSA, dexamethasone, gentamycin, fungizone, fetuin) as previously described^40^. Cells from several donors were pooled and grown at 37 °C in a humidified atmosphere of 5% CO_2_. Differentiation of myoblasts (i.e. activated satellite cells) into myotubes was initiated at ∼80–90% confluence, by switching to α-Minimum Essential Medium with 2% penicillin-streptomycin, 2% FBS, and fetuin (0.5 mg/ml). The medium was changed every other day and cells were grown up to 5 days. For pharmacological treatments, cells were exposed to a PPARα or PPARβ agonist (GW7647 and GW0742, respectively) or a PPARβ antagonist (GSK0660) for 24 h at the end of the differentiation.

### Overexpression of PLIN5 in human myotubes

For overexpression experiments, adenoviruses expressing in tandem GFP and human PLIN5 (hPLIN5) were used (Vector Biolabs, Philadelphia, PA). Control was performed using adenoviruses containing GFP gene only. Myotubes were infected with both adenoviruses at day 4 of differentiation and remained exposed to the virus for 24 h in serum-free DMEM containing 100 μM of oleate complexed to BSA (ratio 2/1). Oleate was preferred to palmitate for lipid loading of the cells, to favor triacylglycerol (TAG) synthesis and to avoid the intrinsic lipotoxic effect of palmitate^41^. As a model of lipid-induced lipotoxicity and insulin resistance, oleate was replaced by palmitate in some experiments to metabolically challenge the cells.

### Animal studies

All experimental procedures were approved by a local ethics committee (CEEA122 INSERM US006/CREFRE, protocol n°C14/U1048/DL/13) and performed according to INSERM animal care facility guidelines and to the 2010/63/UE European Directive for the care and use of laboratory animals. Sixteen week-old male PPARβ knockout and wild-type mice on a SV129/C57Bl6 background were used for muscle tissue collection.

Four-week-old C57BL/6 J male mice were housed in a pathogen-free barrier facility (12 h light/dark cycle) and fed either normal chow diet (10% calories from fat) (D12450J, Research Diets, New Jersey) or high-fat diet (60% calories from fat) (D12492, Research Diets, New Jersey). To induce an *in vivo* knockdown of PLIN5 specifically in skeletal muscle, mice were injected with 1 × 10^11^ GC (i.e. genome copy) of AAV1 vector (Vector Biolabs, Philadelphia, PA) in *tibialis anterior* muscles at 10 weeks of age. Each mouse had one leg injected with AAV1-shPLIN5 and the contralateral leg injected with AAV1-shNT (nontarget) as a control. Six weeks following the injections, mice were killed by cervical dislocation and muscles (i.e. *tibialis anterior* and *extensor digitorum longus*) were dissected and either used *ex-vivo* for palmitate and glucose oxidation assays or stored at −80 °C for protein, RNA and lipid analyses.

### Real-time RT-qPCR

Total RNA from cultured myotubes or *tibialis anterior* muscle was isolated using Qiagen RNeasy mini kit according to manufacturer’s instructions (Qiagen GmbH, Hilden, Germany). The quantity of RNA was determined on a Nanodrop ND-1000 (Thermo Scientific, Rockford, IL, USA). Reverse-transcriptase PCR was performed on a Techne PCR System TC-412 using the Multiscribe Reverse Transcriptase method (Applied Biosystems, Foster City, CA). Real-time quantitative PCR (qPCR) was performed to determine cDNA content. All primers were bought from Applied Biosystems and were: 18 S (Taqman assay ID: Hs99999901_s1), PLIN5 (Hs00965990_m1 and Mm00508852_m1), and PDK4 (Hs01037712_m1). The amplification reaction was performed in duplicate on 10ng of cDNA in 96-well reaction plates on a StepOnePlus^TM^ system (Applied Biosystems). All expression data were normalized by the 2^(ΔCt)^ method using 18 S as internal control.

### Western blot analysis

Muscle tissues and cell extracts were homogenized in a buffer containing 50 mM HEPES, pH 7.4, 2 mM EDTA, 150 mM NaCl, 30 mM NaPPO_4_, 10 mM NaF, 1% Triton X-100, 1.5 mg/ml benzamidine HCl and 10 μl/ml of each: protease inhibitor, phosphatase I inhibitor and phosphatase II inhibitor (Sigma-Aldrich). Tissue homogenates were centrifuged for 25 min at 15,000 *g* and supernatants were stored at −80 °C. A total of 30 μg of solubilized proteins from muscle tissue and myotubes were run on a 4–12% SDS-PAGE (Biorad), transferred onto nitrocellulose membrane (Hybond ECL, Amersham Biosciences), and blotted with the following primary antibodies: PLIN5 (#GP31, Progen), ATGL (#2138, Cell Signaling Technology Inc.), Akt (#4691, Cell Signaling Technology Inc.), pAkt S473 (#4060, Cell Signaling Technology Inc.), pAkt T308 (#2965, Cell Signaling Technology Inc.). Subsequently, immunoreactive proteins were blotted with secondary HRP-coupled antibodies (Cell Signaling Technology Inc.) and revealed by enhanced chemiluminescence reagent (SuperSignal West Femto, Thermo Scientific), visualized using the ChemiDoc MP Imaging System and data analyzed using the ImageLab 4.2 version software (Bio-Rad Laboratories, Hercules, USA). GAPDH (#2118, Cell Signaling Technology Inc.) was used as an internal control.

### Determination of glucose metabolism

Cells were pre-incubated with a glucose- and serum-free medium for 90 min, then exposed to DMEM supplemented with D[U-^14^C] glucose (1 μCi/ml; PerkinElmer, Boston, MA). Following incubation, glucose oxidation was determined by counting of ^14^CO_2_ released into the culture medium. The cells were then solubilized in KOH 30% and glycogen synthesis was determined as previously described^42^. Total glycogen content was determined spectrophotometrically after complete hydrolysis into glucose by the α-amiloglucosidase as previously described^43^.

### Determination of fatty acid metabolism

Cells were pulsed overnight for 18 h with [1-^14^C] oleate (1 μCi/ml; PerkinElmer, Boston, MA) and cold oleate (100 μM) to prelabel the endogenous TAG pool. Oleate was coupled to FA-free BSA in a molar ratio of 5:1. Following the pulse, myotubes were chased for 3 h in DMEM containing 0.1 mM glucose, 0.5% FA-free BSA, and 10 μM triacsin C to block FA recycling into the TAG pool as described elsewhere^44^, in absence or presence of 10 μM forskolin to stimulate lipolysis. For electrical pulse stimulation experiments, cells were chased for 24 h in DMEM containing 1 mM glucose, 0.5% FA-free BSA and 10 μM triacsin C while electrically stimulated by 2 ms pulses at a frequency of 0.1 Hz. TAG-derived FA oxidation was measured by the sum of ^14^CO_2_ and ^14^C-ASM (acid soluble metabolites) in absence of triacsin C as previously described^40^. Myotubes were harvested in 0.2 ml SDS 0.1% at the end of the pulse and of the chase period to determine oleate incorporation into TAG and protein content. The lipid extract was separated by TLC using heptane-isopropylether-acetic acid (60:40:4, v/v/v) as developing solvent. All assays were performed in duplicates, and data were normalized to cell protein content. Palmitate oxidation rate was measured as previously described^43^.

### Tissue-specific [2-^3^H] deoxyglucose uptake *in vivo*

Muscle-specific glucose uptake was assessed in response to an intraperitoneal bolus injection of 2-[1,2-^3^H(N)]deoxy-D-Glucose (PerkinElmer, Boston, Massachusetts) (0.4μCi/g body weight) and insulin (3 mU/g body weight). The dose of insulin was determined in preliminary studies to reach a nearly maximal stimulation of insulin signaling and glucose uptake in all muscle types and metabolic tissues. Mice were killed 30 min after injection and tissues were extracted by precipitation of 2-deoxyglucose-6-phosphate as previously described^45^.

### Determination of neutral lipid and ceramide content

Triacylglycerols and diacylglycerols were determined by gas chromatography, and ceramide and sphingomyelin species by high-performance liquid chromatography-tandem mass spectrometry after total lipid extraction as described elsewhere^45^,^46^.

### Statistical analyses

All statistical analyses were performed using GraphPad Prism 5.0 for Windows (GraphPad Software Inc., San Diego, CA). Normal distribution and homogeneity of variance of the data were tested using Shapiro-Wilk and F tests, respectively. One-way ANOVA followed by Tukey’s post hoc tests and Student’s *t*-tests were performed to determine differences between treatments. Two-way ANOVA and Bonferroni’s post hoc tests were used when appropriate. All values in figures and tables are presented as mean ± SEM. Statistical significance was set at *p* < 0.05.

## Additional Information

**How to cite this article**: Laurens, C. *et al*. Perilipin 5 fine-tunes lipid oxidation to metabolic demand and protects against lipotoxicity in skeletal muscle. *Sci. Rep.* **6**, 38310; doi: 10.1038/srep38310 (2016).

**Publisher's note:** Springer Nature remains neutral with regard to jurisdictional claims in published maps and institutional affiliations.

## Supplementary Material

Supplemental Data
