# Supplementary material for: Perilipin 5 fine-tunes lipid oxidation to metabolic demand and protects against lipotoxicity in skeletal muscle
Source: Sci Rep. 2016 Dec 6;6:38310. doi: 10.1038/srep38310 (PMC5138838; doi:10.1038/srep38310)
Supplement: Supplemental Data [file srep38310-s1.pdf]

SUPPPLEMENTARY INFORMATION

Perilipin 5 fine-tunes lipid oxidation to metabolic demand and protects against lipotoxicity in skeletal muscle

Claire Laurens[1,2], Virginie Bourlier[1,2], Aline Mairal[1,2], Katie Louche[1,2], Pierre-Marie Badin[1,2], Etienne Mouisel[1,2], Alexandra Montagner[2,3], André Marette[4,6], Angelo Tremblay[5,6], John S. Weisnagel[7], Hervé Guillou[2,3], Dominique Langin[1,2,8], Denis R. Joanisse[5,6$], Cedric Moro[1,2$]

[$] These authors contributed equally to this work.

[1]INSERM, UMR1048, Institute of Metabolic and Cardiovascular Diseases, Toulouse, France
[2]University of Toulouse, Paul Sabatier University, France
[3]INRA, UMR 1331, TOXALIM, Toulouse, France
[4]Department of Medicine, Laval University, Quebec City, Canada
[5]Department of Kinesiology, Laval University, Quebec City, Canada
[6]Centre de Recherche de l'Institut Universitaire de Cardiologie et de Pneumologie de Québec, Laval University, Quebec City, Canada
[7]CHU-CHUQ, Laval University, Quebec City, Canada
[8]Toulouse University Hospitals, Department of Clinical Biochemistry, Toulouse, France

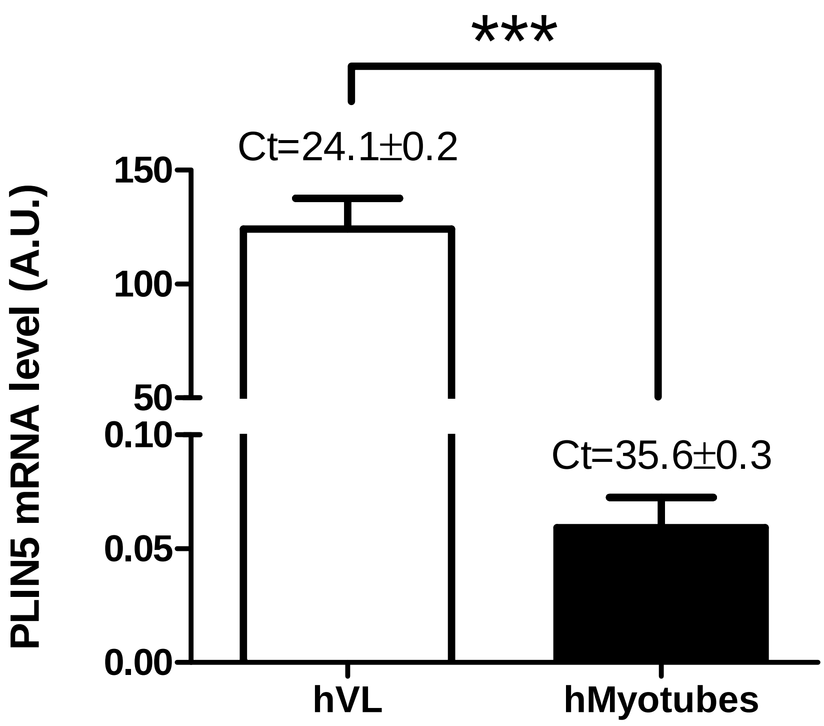

Supplemental Figure S1. PLIN5 gene expression in human native skeletal muscle and cultured myotubes. PLIN5 mRNA levels in human *vastus lateralis* muscle biopsy samples and human primary myotubes (n=9). Average Ct ± SEM are shown on the graph. ***$p<0.001$ versus hVL.

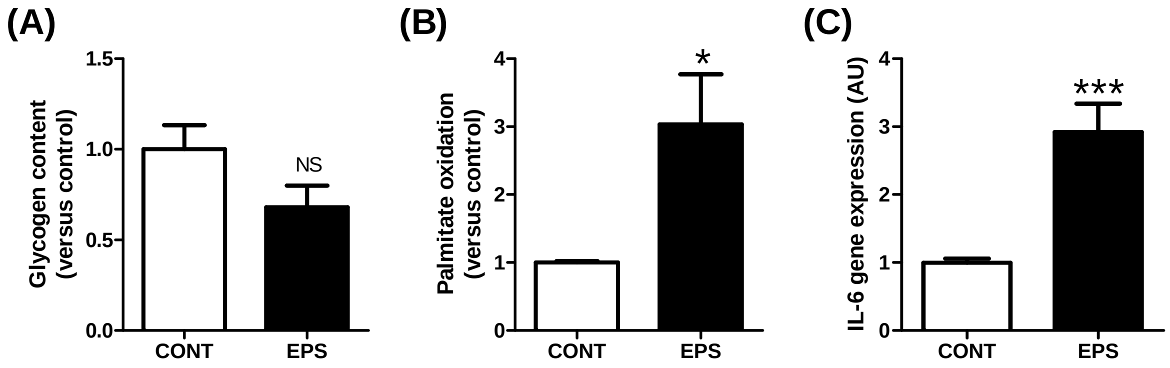

Supplemental Figure S2. Validation of the electrical pulse stimulation model in human myotubes. (**A**) Total glycogen content (n=4), (**B**) palmitate oxidation (n=4) and (**C**) interleukin-6 (IL-6) gene expression (n=4) were measured in control (CONT) and electrically stimulated (EPS) myotubes for 24 hours. NS : non-significant, *p<0.05, ***p<0.001.

(A)

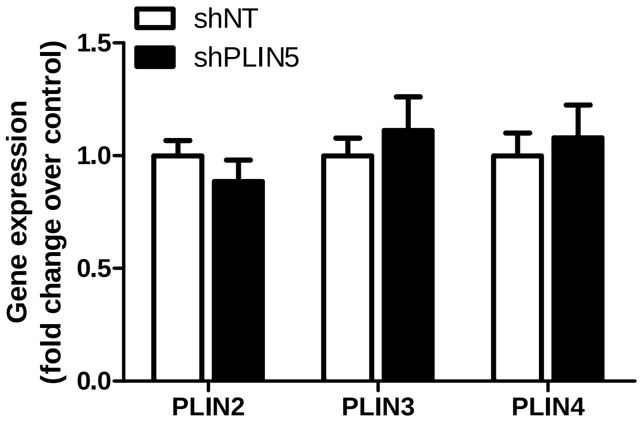

(B)

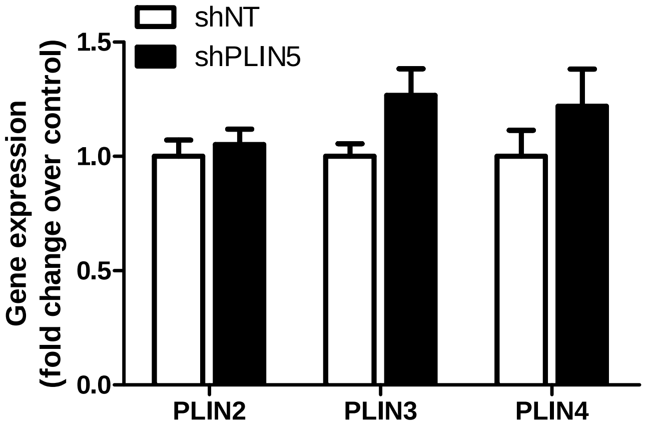

Supplemental Figure S3. PLIN5 knockdown does not induce any compensatory changes in other PLIN isoforms. PLIN2, PLIN3 and PLIN4 gene expression in control (shNT) and PLIN5 knocked down (shPLIN5) *tiabialis anterior* muscles, measured in mice fed either (**A**) normal chow (NCD) or (**B**) high-fat diets for 12 weeks (n=6).

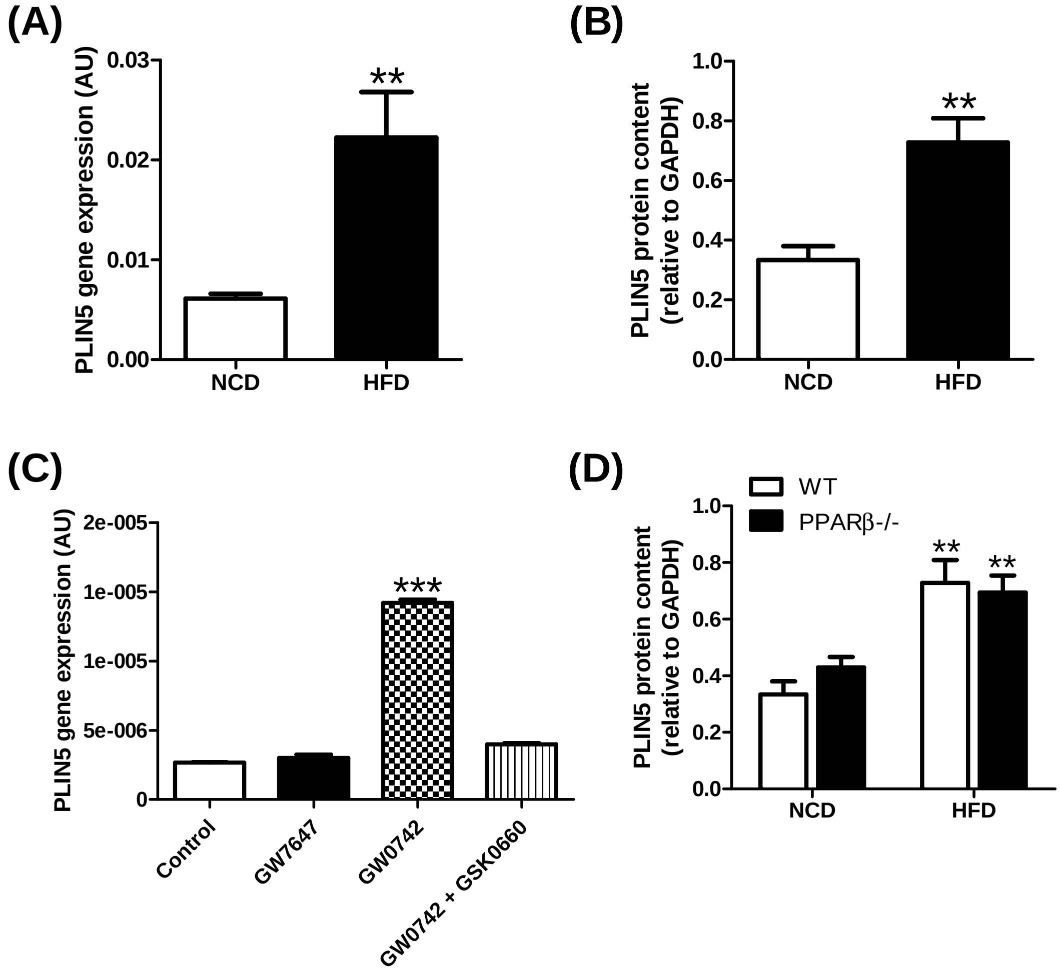

Supplemental Figure S4. PLIN5 is induced by high-fat feeding in mouse skeletal muscle independently of PPARβ activation. PLIN5 (**A**) gene expression and (**B**) protein content were measured in skeletal muscle of mice fed either normal chow (NCD) or high-fat (HFD) diet for 12 weeks (n=7). (**C**) PLIN5 gene expression was measured in myotubes treated for 24 h in absence (control) or presence of selective PPARα agonist GW7647 1 nM, PPARβ agonist GW0742 1nM and PPARβ antagonist GSK0660 500 nM (n=3). (**D**) PLIN5 protein content was measured in skeletal muscle from wild-type (WT) and PPARβ knockout (PPARβ-/-) mice fed either chow (NCD) or high-fat (HFD) diet (n=6). **p<0.01, ***p<0.001 versus control.
